# A spin promotion effect in catalytic ammonia synthesis

**DOI:** 10.1038/s41467-022-30034-y

**Published:** 2022-05-02

**Authors:** Ang Cao, Vanessa J. Bukas, Vahid Shadravan, Zhenbin Wang, Hao Li, Jakob Kibsgaard, Ib Chorkendorff, Jens K. Nørskov

**Affiliations:** Department of Physics, Technical University of Denmark, 2800 Kongens Lyngby, Denmark

**Keywords:** Heterogeneous catalysis, Density functional theory

## Abstract

The need for efficient ammonia synthesis is as urgent as ever. Over the past two decades, many attempts to find new catalysts for ammonia synthesis at mild conditions have been reported and, in particular, many new promoters of the catalytic rate have been introduced beyond the traditional K and Cs oxides. Herein, we provide an overview of recent experimental results for non-traditional promoters and develop a comprehensive model to explain how they work. The model has two components. First, we establish what is the most likely structure of the active sites in the presence of the different promoters. We then show that there are two effects dictating the catalytic activity. One is an electrostatic interaction between the adsorbed promoter and the N-N dissociation transition state. In addition, we identify a new promoter effect for magnetic catalysts giving rise to an anomalously large lowering of the activation energy opening the possibility of finding new ammonia synthesis catalysts.

## Abstract (web summary)

Catalytic ammonia synthesis continues to attract significant interest. Here, the authors develop a model to explain a large body of recent experimental data on new promoters. They find new promotion mechanism mediated by coupling between adsorption and spin polarization on the surface atoms of magnetic catalysts.

## Introduction

Ammonia plays an important role as the basis for nitrogen fertilizer production^1,2^, and is also being considered as a potential energy carrier in a sustainable future^3,4^. The Haber–Bosch process is the cornerstone in today’s ammonia synthesis^5^, and is likely to continue to be important even as the source of hydrogen shifts from natural gas steam reforming to electrolysis in a new era based on sustainable electricity production. An efficient process at mild conditions would be extremely useful. It could facilitate decentralization and compatibility with small-scale green-hydrogen production units.

Alternatives to the Haber–Bosch process include photochemical^6^, electrochemical^7^, thermal looping^8^, and plasma^9^ processes, but these interesting options are still some way into the future. The current commercial catalyst for the Haber–Bosch process is a structurally (with Al_2_O_3_) and electronically (mainly with K_2_O) promoted iron-based catalyst, quite similar to the one developed by Mittasch in the early twentieth century^10^. In the last decade of the twentieth century, a carbon-supported ruthenium-based catalyst (promoted with Ba and K)^11^ was developed as a substitute for iron-based catalyst in the Kellogg Advanced Ammonia Process^12^. However, this so-called “second-generation” ammonia catalyst could not take over the classic iron-based catalysts mainly because of the relatively high cost of Ru.

## Results

### Summary of recent experimental data

**Fig. 1:**
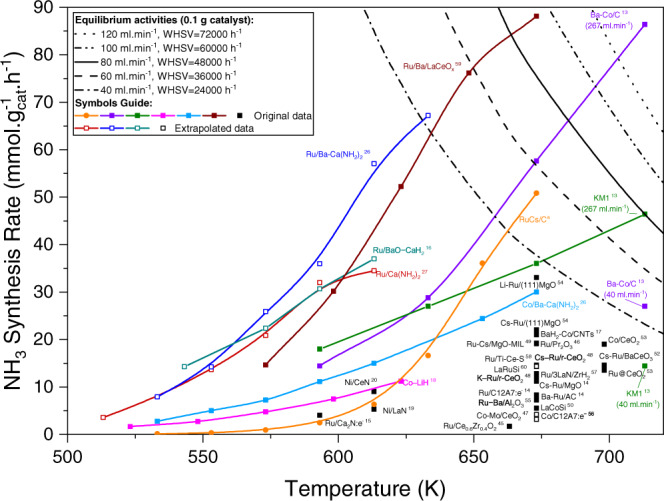
Experimental activities for ammonia synthesis. Overview of some of the most recent and promising catalysts reported for ammonia synthesis^13–20,26,27,45–60^. Most of the data points (filled symbols) are extracted from original references based on the following conditions: pressure = 10 bar, H_2_/N_2_ = 3. The open symbols are estimated values (at 10 bar) based on the original data reported at slightly lower pressures (9 bar). The curves in the upper right corner show the equilibrium activity over 0.1 g of catalyst in different overall flowrates (all H_2_/N_2_ = 3). Data from the industry-standard, the K-promoted Fe catalyst (KM1 from Haldor Topsøe)^13^ is included as a benchmark. The references for these catalysts are marked with superscripts. Superscript “a” indicates the prepared RuCs/C catalyst in this work.

A number of new catalysts and promoters have appeared in the literature, and we have summarized ammonia synthesis data for some of the most recent, active materials in Fig. 1 and Supplementary Table 1. We focus on metals that are less reactive (weaker N bonding) than Fe with different “super”-promoters. For example, carbon-supported Co catalysts have been promoted with Barium^13^. Inspired by the significant electronic promoting effects of “electron-donating” materials on the activity of Ru- and Fe-based catalysts, researchers started to investigate the promoting effects of electrides (e.g., [Ca_24_Al_28_O_64_]^4+^(4e^−^)^14^, Ca_2_N:e^−^^15^). Two other classes of materials have gained interest for ammonia synthesis recently: hydrides (especially alkali and alkaline earth metals) and amides (alkaline earth metals)^16–18^. Different transition metals have been used together with these amides to make active catalysts for NH_3_. The most recent development in the field is a report on using rare-earth metal nitrides (e.g., LaN and CeN) as support for Ni^19,20^.

The effect of K and Cs as a promoter for Fe and Ru catalysts is well described^21–24^, but the rest of the data in Fig. 1 pose a number of questions:

- How do alkaline earth metals like Ba and Ca work as a promoter? It has been suggested that BaO acts as a structural promoter for Ru^25^. However, that does not explain how Ba makes a very unreactive metal like Co active.
- What is the role of amides? They are proposed to have both structural (e.g., shape control of Ru nanoparticles) and electronic (i.e., strong electron donation abilities) promotion effects^26,27^. Are they different from the oxides or hydrides of the same promoter?
- What is the effect of Li compounds? It has been proposed that LiH acts as a reducing agent removing activated N atoms from the transition metal site^18^.
- What is the role of electrides? It has been explained^14,28^ as an electrostatic interaction analogous to the effect of alkali adsorption.
- How do La and Ce act as a promoter? It has been proposed that N_2_ activation takes place over LaN sites and provides activated N while Ni acts as H_2_-activator^19^.

The spectrum of explanations is quite broad. In the following we propose a comprehensive model for these effects.

The starting point for our analysis is the current understanding of the ammonia synthesis mechanism and structure of the active site for the process. For the traditional ammonia catalysts, N_2_ dissociation is found to be rate determining^1,2,29^, and theoretical analysis suggests that to be the case for all but the most reactive metals (those to the left of Fe in the periodic table)^30^. Another key finding is a very strong structure sensitivity where step-like structures are responsible for the activation and dissociation of the N_2_ molecule^31^. Alkali metal promoters for Fe- and Ru-based catalysts are well studied experimentally^1,21,22^ and the effect has been described theoretically as a stabilization of the N_2_ transition state (TS) for dissociation in combination with a destabilization of NH_*x*_ intermediates due to an electrostatic interaction with the dipole field setup by the adsorbed alkali atom which transfers its electron to the surface^23,24^.

### Structure of the promotor phase

**Fig. 2:**
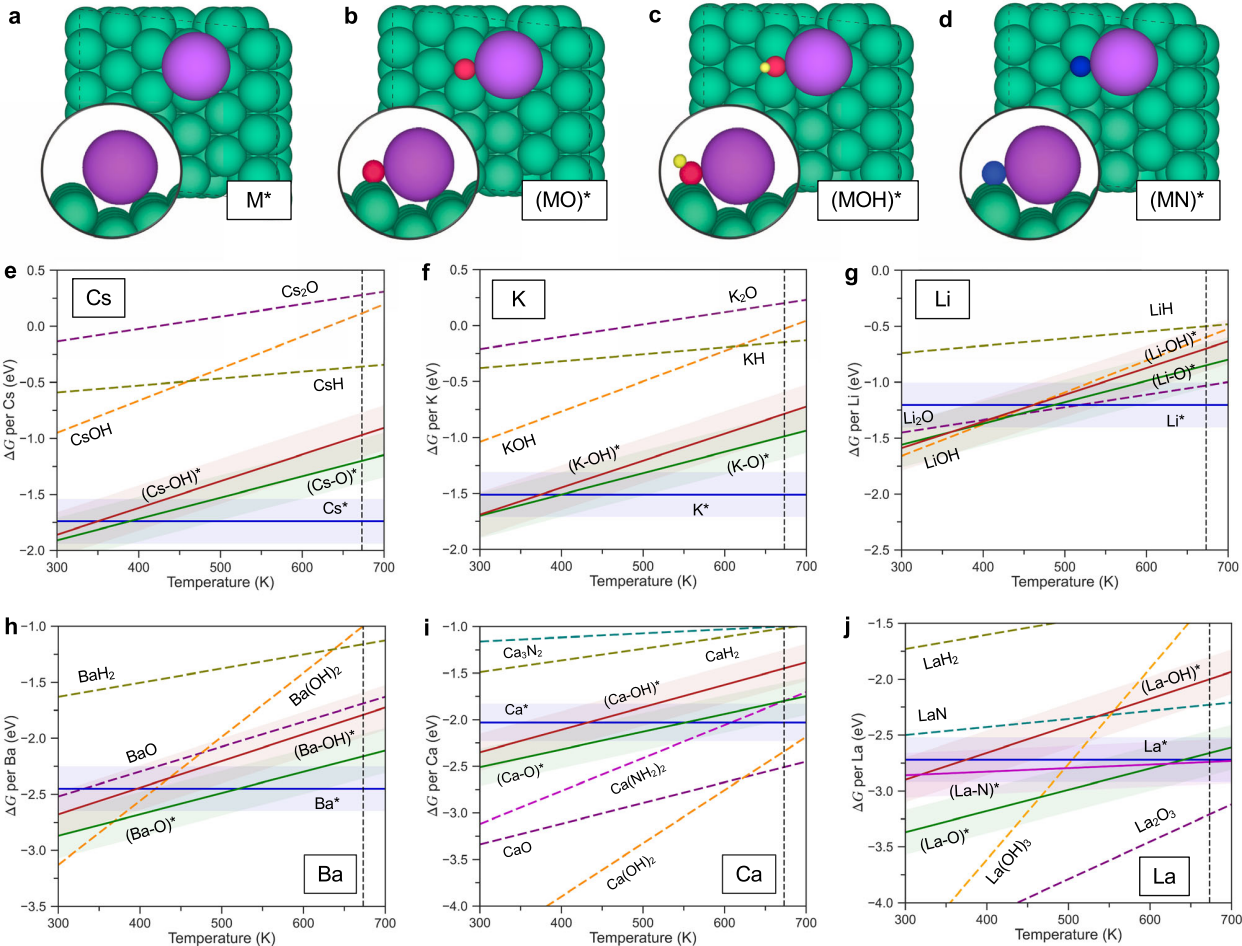
Surface phase diagrams. **a**–**d** Top and side view of adsorption structures of M*/(MO)*/(MOH)*/(MN)* on the Ru($10\bar{1}5$) surface. M means metallic promoter atom. Green, purple, red, yellow, and blue spheres represent Ru, promoter M, oxygen, hydrogen, and nitrogen atoms. **e**–**j** Phase diagrams of Cs, K, Li, Ba, Ca and La promoted Ru in equilibrium with their oxides, hydroxides hydrides, nitrides or amides under reaction conditions. Bulk species are shown as dashed lines, while adsorbed species are shown as full lines and identified by a *. A typical DFT uncertainty of ±0.2 eV is shown shaded for the adsorbed species. The reaction conditions are *T* = 673 K, H_2_ = 7.5 bar, NH_3_ = 0.1 bar (N_2_ conversion of 2%), P_H2O_ = 10^−7^ bar, chosen to simulate an extremely dry reactant gas. All data used in the figure can be found in Supplementary Tables 2–4.

To establish our model, we firstly discuss the nature of the active site including the promoter, and next we provide an understanding of how the different promoters work. The catalysts in Fig. 1 are named after the precursor compounds used in the synthesis of the catalyst. This does not necessarily describe the structure of the catalyst under reaction conditions. To understand the structure of the active sites during ammonia synthesis, we did extensive density functional theory (DFT) calculations (see Methods section for details) of the surface phase diagrams for hexagonal Ru($10\bar{1}5$) and Co($10\bar{1}5$) stepped surfaces. The surface structures considered and the free energy diagrams for different promoters on the two surfaces are shown in Fig. 2 and Supplementary Figs. 1 and 2. All surface species are assumed to be in quasi-equilibrium with gas phase H_2_, NH_3_ and H_2_O. This implicitly assumes that N_2_ dissociation is rate limiting for these weak-bonding metals in agreement with previous analysis of the process. As noted above, it takes a very strong bonding catalyst for the hydrogenation steps to become rate limiting^2,32^. Some reports suggest that some of the non-traditional promoters give reaction orders in N_2_ that are less than one indicating another rate determining step^14,18,26^, but the analysis is most likely incomplete—our analysis of the same experimental data shows a reaction order of one for N_2_ (Supplementary Fig. 3).

Figure 2 shows that under reaction conditions, the oxide, hydride, nitride and amide forms of K, Cs, Li, and Ba are reduced out of the bulk precursor migrating to the step sites of the catalyst (Ru in this case) metal particles (structure see Fig. 2a–d). There will still be an oxide (or other precursor) phase of the promoter present, which may dominate any experimental analysis of the system, but our theoretical analysis indicates that the strong bonding of the promoter atoms to the step-like active sites on the host catalyst allows some promoter atoms to be reduced out and form the catalytically active phase. This means that the nature of the precursor is likely to have a minor effect on the nature of the active site. It can, however, affect the number of active sites. The picture is essentially the same for Co-based catalysts, except that it may be somewhat harder to reduce out the precursors in the presence of Co since it binds the precursor atoms a little weaker than Ru. In this case the nature of the precursor can therefore have an effect. If the water content is higher, it becomes more difficult to reduce out the precursor (Supplementary Fig. 4). For Ca and La, it is difficult to reduce the oxide and hydroxides except at the lowest water content considered. The nitride, hydride and amide forms used in recent experiments^19,20^ are, however, still reducible at these conditions, if the promoter goes to a step site of the catalyst. We have not considered the stability of electrides in this study, but find it likely that these compounds, which typically contain alkaline earth elements, can also be partially reduced to provide promoters at the active sites of the transition metal.

### Promotion mechanisms

**Fig. 3:**
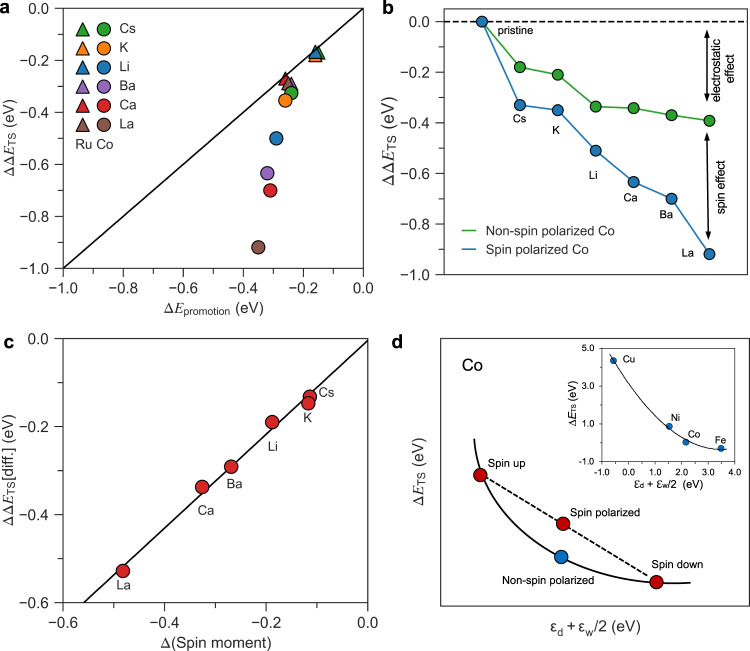
Electrostatic and magnetic effects. **a** The N-N transition state (TS) energy stabilization (ΔΔ*E*_TS_ = Δ*E*_TS_(with promoter) – Δ*E*_TS_(without promoter)) for different promoters as a function of the electrostatic promotion (Δ*E*_promotion_ = –μ_N-N_ ℇ_promoter_). Triangles and squares refer to the Ru and Co surface, respectively. **b** ΔΔE_TS_ for different promoters for spin-polarized and non-spin-polarized Co. **c** The difference ΔΔE_TS_ [diff] = ΔΔE_TS_ (spin polarized) – ΔΔE_TS_ (non-spin polarized) between the TS energy for spin-polarized (blue in **b**) and non-spin-polarized (green in **b**) Co plotted as a function of the promoter-induced change in spin moment of the Co atoms at the active site (Supplementary Table 9). **d** Schematic plot of the relation between ΔE_TS_ and the top of the d band on a spin-polarized and non-spin-polarized surface Inset shows our calculated ΔE_TS_ on different non-spin-polarized late 3d metals as a function of the top of the d band taken from Ref. ^30^ displaying a nonlinear dependence for the late 3d metals.

We now turn to the way promoters work to enhance the rate of ammonia synthesis. Since we are only considering weak-bonding catalysts, that is, metals bonding nitrogen weaker than Fe, an enhancement of the rate will primarily come from a lowering of the TS energy for N_2_ dissociation, which is the focus in the following. As mentioned above, electrostatic effects go far in explaining the promoting effect of Cs and K^23,24^. We suggest that such an electrostatic effect is also operative for Li, Ba, and Ca. We also include a rare-earth metal, La, to illustrate the generality of the effect. Figure 3a compares the calculated promotion effect on the TS energy for N_2_ dissociation with an estimate of the electrostatic dipole interaction between the promoter-induced electrical field, ℇ_promoter_, and the dipole moment of the TS, μ_N-N_ (see Supplementary Figs. 5–8 and Supplementary Tables 5–8 for details):

$$\varDelta {E}_{{{{{{\rm{promotion}}}}}}}=-{\mu }_{{{{{{\rm{N}}}}}}-{{{{{\rm{N}}}}}}}{\varepsilon }_{{{{{{\rm{promoter}}}}}}}$$

For Ru this describes the trends and the absolute value of the effect quite well, both for the traditional alkali promotion and for Li, Ba, Ca and La. For Co this is different. Here the effect of K and Cs is still quite well described by the electrostatic model, but clearly there is an extra effect in play here (Fig. 3b).

We identify the extra promotion by Li, Ba, Ca, La on Co as related to the spin polarization of Co (Supplementary Figs. 9–12). The non-traditional promoters reduce the spin polarization of the neighboring Co atoms defining the active site for N_2_ dissociation, and the extra promotion effect is directly proportional to the promoter-induced reduction in spin moment of the Co atoms, see Fig. 3c.

To understand this effect, we first point out that it has been found previously that the interaction of several adsorbates with a spin-polarized surface is less exothermic than on the non-polarized counterparts and that adsorption reduces the spin moment of the surface^33,34^. The calculated N-N TS on the non-spin-polarized Co is for example found to be ~1 eV lower in energy than that for spin-polarized Co. The weaker coupling between the spin-polarized surface and the N-N TS for Co can be viewed as an effect of a nonlinear dependence of the TS energy on the d band position for the 3d metals (Fig. 3d). Splitting the d bands into a spin-up and spin-down component gives an average adsorption energy that is less negative than the non-spin-polarized version. Separate energy contributions from the majority and minority spin channels were also considered in the model proposed by Bhattacharjee et al.^33^.

Figure 3 shows two effects which together give the anomalous spin promotion effect of Li, Ba, Ca and La. First, these promoters reduce the spin moment of the surface the most. Second, since the spin moment has already been reduced by the promoter, the reduction of the N-N TS energy by spin polarization is reduced. This gives an indirect attractive interaction between the promoter and the TS. We find the spin promotion effect to work for other magnetic metals as well (Supplementary Fig. 13 and Supplementary Table 5), and we have thus discovered a new promotion effect working only for magnetic materials.

**Fig. 4:**
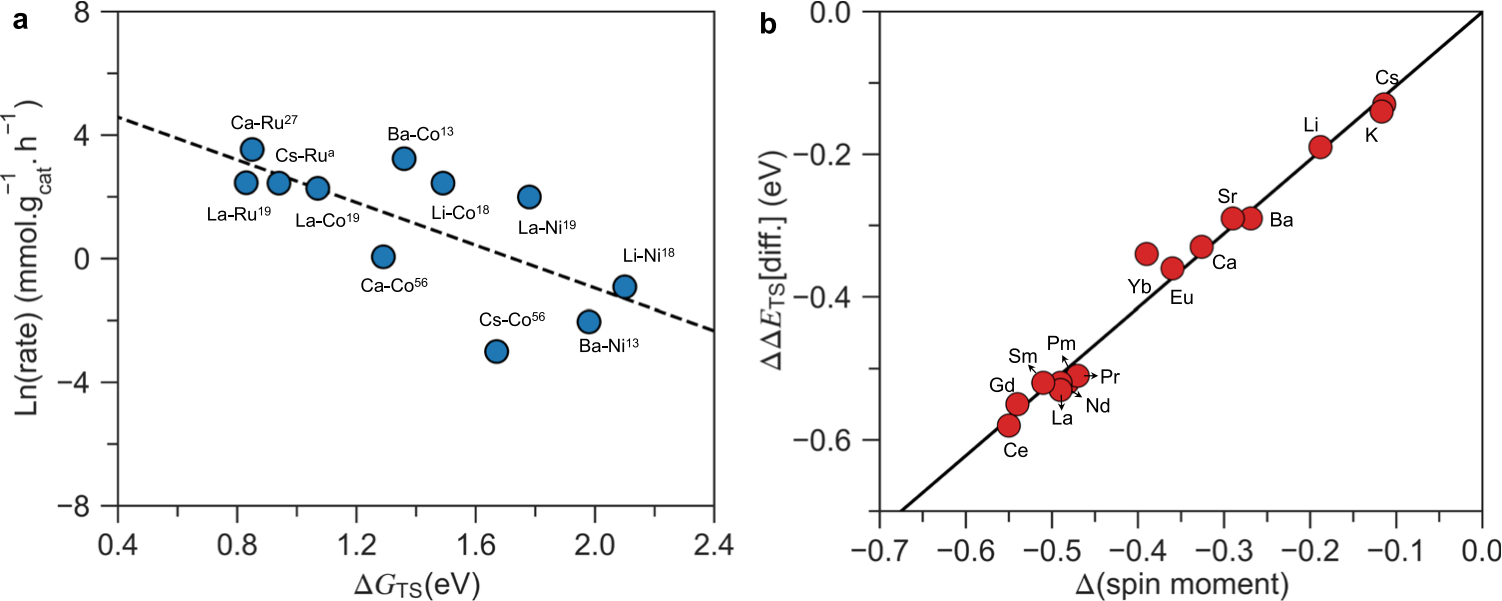
The application of the proposed spin effect. **a** The experimental activity as a function of calculated transition state free energy. The references for these catalysts are marked with superscripts. Superscript “a” indicates the prepared RuCs/C catalyst in this work. **b** The difference ΔΔE_TS_ [diff] = ΔΔE_TS_ (spin polarized) – ΔΔE_TS_ (non-spin polarized) between the TS energy for spin-polarized and non-spin-polarized Co plotted as a function of the promoter-induced change in spin moment of the Co atoms at the active step site.

The model outlined above describes the many different promoters and transition metal catalysts quite well. By plotting the experimental ammonia synthesis rate for the different catalysts in Fig. 1 against the calculated TS free energy for the different catalysts and promoters, as shown in Fig. 4a (see Supplementary Table 6 for details), we observe a good description of the trends, especially considering that the experimental data are not normalized per surface atom. We note that the trends need not be linear since both the TS energy and the energy of intermediates change with promotion.

Our model resolves the long-standing question of how Ba and Ca work as a promoter. For Ru and other non-magnetic catalysts, the effect is primarily electrostatic—the electropositive atoms transfer electrons to the surface and set up an electrical field stabilizing the TS of N_2_ dissociation. This effect is completely analogous to the way K and Cs promoters work—and the effect is of the same order of magnitude. Li has a similar effect. The most remarkable effect that the present model explains is the extraordinary promotion of Co by Li, Ba, Ca and La. Co is normally quite inert toward N_2_ dissociation and shows minor ammonia synthesis activity even when promoted by alkalis.

The new spin promotion effect is not limited to the promoters considered so far and opens to possibility of new promoter systems for magnetic catalysts. Figure 4b summarizes changes in the TS energy for N_2_ dissociation over Co for a number of promoters. It should be noted, however, that stability of the promoter at the active site is as important as the spin reduction effect, as pointed out above. Most promoters are very oxyphilic and the gas feed needs to be extremely dry in order to be able to reduce out the promoter. In addition, nitride phases are often competing with the promoter phases, in particular at high ammonia concentration as illustrated in Supplementary Fig 14.

## Methods

### Reaction order calculation method

Correction of calculated reaction orders for Ru/C12A7:e^−^ catalyst reported by Kitano et al.^14^.

The method for calculating reaction orders as explained in the original paper:

Kitano et al. calculated the reaction orders by assuming a power law rate expression (${{{{{{\rm{r}}}}}}}_{{{{{{{\rm{NH}}}}}}}_{3}}={{{{{\rm{K}}}}}}{{{{{{\rm{P}}}}}}}_{{{{{{{\rm{N}}}}}}}_{2}}^{{{{{{\rm{\alpha }}}}}}}{{{{{{\rm{P}}}}}}}_{{{{{{{\rm{H}}}}}}}_{2}}^{{{{{{\rm{\beta }}}}}}}{{{{{{\rm{P}}}}}}}_{{{{{{{\rm{NH}}}}}}}_{3}}^{{{{{{\rm{\gamma }}}}}}}$), then at constant temperature:

- For ammonia reaction order: ${{{{{{\rm{P}}}}}}}_{{{{{{{\rm{N}}}}}}}_{2}}$ and ${{{{{{\rm{P}}}}}}}_{{{{{{{\rm{H}}}}}}}_{2}}$ were kept constant and the total flowrate was changed to get the relation between different ${{{{{{\rm{P}}}}}}}_{{{{{{{\rm{NH}}}}}}}_{3}}$ and ${{{{{{\rm{r}}}}}}}_{{{{{{{\rm{NH}}}}}}}_{3}}$.
- For nitrogen reaction order: ${{{{{{\rm{P}}}}}}}_{{{{{{{\rm{H}}}}}}}_{2}}$ and total flowrate were kept constant, then α was calculated from the slope of ${{\log }}\left({{{{{{\rm{r}}}}}}}_{{{{{{{\rm{NH}}}}}}}_{3}}\right)$ and ${{\log }}\left({{{{{{\rm{P}}}}}}}_{{{{{{{\rm{N}}}}}}}_{2}}\right)$.
- For hydrogen reaction order: ${{{{{{\rm{P}}}}}}}_{{{{{{{\rm{N}}}}}}}_{2}}$ and total flowrate were kept constant, then β was calculated from the slope of ${{\log }}\left({{{{{{\rm{r}}}}}}}_{{{{{{{\rm{NH}}}}}}}_{3}}\right)$ and ${{\log }}\left({{{{{{\rm{P}}}}}}}_{{{{{{{\rm{H}}}}}}}_{2}}\right)$.

The fundamental issue with Kitano et al. calculations:

Assuming power law as the rate expression, to get reaction order for each component, it is basically needed to measure the dependence of reaction rate to that component’s pressure while keeping all others’ pressure constant. However, this may not be always possible due to technical limitations. For example, according to Kitano et al. method, for measurements on calculating N_2_ order, both N_2_ and NH_3_ pressures were changing because the total flowrate was kept constant.

With just keeping ${{{{{{\rm{P}}}}}}}_{{{{{{{\rm{H}}}}}}}_{2}}$ constant, the power law rate becomes ${{{{{{\rm{r}}}}}}}_{{{{{{{\rm{NH}}}}}}}_{3}}={{{{{{{\rm{K}}}}}}}^{{\prime} }} {{{{{{\rm{P}}}}}}}_{{{{{{{\rm{N}}}}}}}_{2}}^{{{\alpha }}}{{{{{{\rm{P}}}}}}}_{{{{{{{\rm{NH}}}}}}}_{3}}^{{{\gamma }}}$ (with ${{{{{{\rm{K}}}}}}}^{{\prime} }={{{{{\rm{K}}}}}}{{{{{{\rm{P}}}}}}}_{{{{{{{\rm{H}}}}}}}_{2}}^{{{\beta }}}$). Then, by taking logarithm of each side of the equation, it becomes ${{{{{\rm{log }}}}}}\left({{{{{{\rm{r}}}}}}}_{{{{{{{\rm{NH}}}}}}}_{3}}\right)-{{\gamma }}{{{{{\rm{log }}}}}}\left({{{{{{\rm{P}}}}}}}_{{{{{{{\rm{NH}}}}}}}_{3}}\right)={{{{{\rm{log }}}}}}\left({{{{{{\rm{K}}}}}}}^{{\prime} }\right)+{{\alpha }}{{{{{\rm{log }}}}}}\left({{{{{{\rm{P}}}}}}}_{{{{{{{\rm{N}}}}}}}_{2}}\right)$. Based on Figure 4b in Kitano et al. paper, the N_2_ order was calculated as the slope of ${{{{{\rm{log }}}}}}\left({{{{{{\rm{r}}}}}}}_{{{{{{{\rm{NH}}}}}}}_{3}}\right)$ and ${{{{{\rm{log }}}}}}\left({{{{{{\rm{P}}}}}}}_{{{{{{{\rm{N}}}}}}}_{2}}\right)$. However, according to the derivation of power law rate at constant ${{{{{{\rm{P}}}}}}}_{{{{{{{\rm{H}}}}}}}_{2}}$, the N_2_ order should have been calculated from the slope of ${{{{{\rm{log }}}}}}\left({{{{{{\rm{r}}}}}}}_{{{{{{{\rm{NH}}}}}}}_{3}}\right)-{{\gamma }}{{{{{\rm{log }}}}}}\left({{{{{{\rm{P}}}}}}}_{{{{{{{\rm{NH}}}}}}}_{3}}\right)$ and ${{{{{\rm{log }}}}}}\left({{{{{{\rm{P}}}}}}}_{{{{{{{\rm{N}}}}}}}_{2}}\right)$. The same principles should be also applied for correct calculation of H_2_ order.

Procedure for calculation of correct N_2_ and H_2_ orders:

The step-by-step procedure for calculating the correct reaction order for Ru/C12A7:e^–^ catalyst reported by Kitano et al. is explained below:

1. ${{\log }}\left({{{{{{\rm{r}}}}}}}_{{{{{{{\rm{NH}}}}}}}_{3}}\right)$ values were extracted from Figure 4b of the original paper.
2. ${{{{{{\rm{r}}}}}}}_{{{{{{{\rm{NH}}}}}}}_{3}}$ values were calculated from the extracted data.
3. Total molar rate of the gas in the system was calculated using the reported testing conditions.
4. Ammonia mole fraction was calculated by the ratio of $\frac{{{{{{{\rm{r}}}}}}}_{{{{{{{\rm{NH}}}}}}}_{3}}}{{{{{{{\rm{system}}}}}}}^{{\prime} }{{{{{{\rm{s}}}}}}\; {{{{{\rm{total}}}}}}\; {{{{{\rm{molar}}}}}}\; {{{{{\rm{rate}}}}}}}}$.
5. ${{{{{{\rm{P}}}}}}}_{{{{{{{\rm{NH}}}}}}}_{3}}$ was calculated based on ammonia mole fraction and the system’s total pressure.
6. ${{{{{\rm{\gamma }}}}}}{{\log }}\left({{{{{{\rm{P}}}}}}}_{{{{{{{\rm{NH}}}}}}}_{3}}\right)$ was calculated using *γ* provided in Table S3 of the original paper’s SI.
7. ${{\log }}\left({{{{{{\rm{r}}}}}}}_{{{{{{{\rm{NH}}}}}}}_{3}}\right)-{{{{{\rm{\gamma }}}}}}{{\log }}\left({{{{{{\rm{P}}}}}}}_{{{{{{{\rm{NH}}}}}}}_{3}}\right)$ values were calculated for each data point.

### Experimental method

RuCs/C catalyst was prepared by incipient wetness method using high surface area carbon (PBX-51) as support. Ruthenium nitrosyl nitrate solution in dilute nitric acid (1.5 wt%, Sigma-Aldrich) and cesium carbonate (99.995% trace metal basis, Sigma-Aldrich) were used as Ru and Cs precursors, respectively. The carbon supported was first impregnated with Ru precursor solution; and after being dried in Ar at 473 K, it was impregnated with Cs precursor solution and dried in Ar flow at 673 K. The final sample was then reduced in hydrogen flow at 673 K prior to activity measurements.

The activity measurements were done in a tubular fixed bed reactor using 100 mg of the sized catalyst particles. The catalyst bed was supported by a layer of stainless steel wool on each side of the bed. Reactant gas contained H_2_/N_2_ with stoichiometric ratio (3/1) and the total flow was adjusted to 80 mL/min. The gas mixtures from the setup were analyzed by two (quadrupole and Time-of-Flight) mass spectrometers.

We perform our experiments in ultra-high pure gases and an oxygen-free system. In line with this, we do not use quartz-wool (i.e., standard conventional material to support catalyst beds in flow reactors) as it contains oxygen (SiO_2_). Instead of quartz-wool, we use stainless steel wool (i.e., same material as our reactor tubes) which is very inert. We did not observe any ammonia produced from the steel wool under our reaction conditions (at 10 bar and ≤400 °C).

### Density functional theory (DFT) calculation method

All DFT calculations were performed using the Vienna Ab initio Simulation Package^35^, employing the generalized gradient approximation^36^ with the Revised PBE functional^37^. Valence electrons were described by the plane-waves with an energy cutoff of 450 eV, whereas core electrons were represented by projector augmented-wave pseudopotentials^38^.

For bulk and all surface calculations, Monkhorst-Pack k-point grid^39^ of 12 × 12 × 12 and 2 × 2 × 1 was used. A lattice constant optimization was performed on the HCP bulk structure of Ru and Co. The ($10\bar{1}5$) surface was generated using four-layer 4 × 6 cells to represent the stepped surface on Co and Ru based on our previous models^23^. The resulting unit cell had six by four surface atoms and included two steps per unit cell. Here we choose the B-type step in our calculations since the B5-site on the B-type step was designated to be the active sites for ammonia synthesis^40^, while no B5-site was present on the A-type step. 15 Å of vacuum separated the slabs in the z-direction, and dipole correction was applied. The bottom two layers of each slab were constrained to their original positions, while the upper layers were allowed to relax. All slabs and bulk were relaxed until all forces converged to less than 0.05 eV. The electronic energy convergence criterion was 10^–5^ eV.

TS of the reactions were located by the climbing image nudged elastic band method^41^ with at least five images generated between the initial and final states. The initial state is putting N_2_ at the fourfold site, and the final state is placing two adsorbed nitrogen atoms around the B5-site in the unit cell, one at the upper step, and one at the lower step, and then perform a geometric relaxation. The TS structures obtained by this method were further refined until the forces on atomic centers reach 0.05 eV/Å. Zero-point energies and entropic contributions were calculated within the harmonic approximation. Free energy corrections of gas-phase species were obtained using the Shomate equation^42^.

The formation energy of adsorbed species (M*/O*/OH*/H*/N*) on the metal surface was calculated by

1 $$\varDelta E({{{{{\rm{species}}}}}})=E({{{{{\rm{slab}}}}}}+{{{{{\rm{M}}}}}}+{{{{{{\rm{H}}}}}}}_{x}{{{{{{\rm{O}}}}}}}_{y}{{{{{{\rm{N}}}}}}}_{z})-E({{{{{\rm{slab}}}}}})-E({{{{{\rm{M}}}}}})-x{E}_{{{{{{\rm{H}}}}}}}-y{{{{{{\rm{E}}}}}}}_{{{{{{\rm{O}}}}}}}-z{E}_{{{{{{\rm{N}}}}}}}.$$

where *E*(M+H_*x*_O_*y*_N_*z*_) and *E*(slab) mean the electronic energy of species (M+H_*x*_O_*y*_N_*z*_) adsorbed on the metal surface and the electronic energy of the pristine metal surface, respectively. *E*(M) is the electronic energy of a single promoter M atom, which refers to the bulk energy of promoter M. *E*_H_ = 0.5*E*_H2_, *E*_O_ = *E*_H2O_ – *E*_H2_, and *E*_N_ = *E*_NH3_ – 1.5*E*_H2_ are relative to the respective gas-phase energies, and *x*, *y*, and *z* are chosen to represent the number of hydrogen, oxygen, and nitrogen atoms in the adsorbed intermediate. H_2_ gas phase values were corrected by adding 0.09 eV as described in Ref. ^43^.

The formation energy of bulk (MH_*x*_O_*y*_N_*z*_) for per M (Δ*E* (bulk)) from experimental values^44^.

The adsorption energy of N* is calculated by

2 $$\varDelta {E}_{{{{{{\rm{N}}}}}}}=E({{{{{\rm{slab}}}}}}+{{{{{\rm{N}}}}}})-E({{{{{\rm{slab}}}}}})\mbox{--}0.5{E}_{{{{{{\rm{N}}}}}}2}$$

where *E*(slab+N) and *E*(slab) mean the total energy of N adsorbed on Ru surface and pristine surface, respectively. *E*_N2_ means the energy of the N_2_ gas phase.

The energy barrier of the N-N TS is calculated by

3 $$\varDelta E({{{{{\rm{TS}}}}}})=E({{{{{\rm{slab}}}}}}+{{{{{\rm{TS}}}}}})-E({{{{{\rm{slab}}}}}})-{E}_{{{{{{\rm{N}}}}}}2}.$$

where *E*(slab+TS) and *E*(slab) mean the total energy of the N-N TS adsorbed on the surface and pristine surface, respectively. *E*_N2_ means the energy of the N_2_ gas phase.

The free energy (Δ*G*) is given by

4 $$\varDelta G=\varDelta H-T\varDelta S=\varDelta E+\varDelta {E}_{{{{{{\rm{zpe}}}}}}}+{\int }_{0}^{T}CpdT-T\varDelta S$$

where Δ*E* means Δ*E* (species), Δ*E* (bulk), or Δ*E* (TS). *E*_ZPE_ is the zero-point energy correction, ΔH is the enthalpy correction, ΔS is the entropy change, Cp is heat capacity, and T is the absolute temperature.

we plotted ∆*E*_promotion_ as a function of the quantity

5 $$\varDelta {E}_{{{{{{\rm{promotion}}}}}}}=-{\mu }_{{{{{{\rm{N}}}}}}-{{{{{\rm{N}}}}}}}{\varepsilon }_{{{{{{\rm{promoter}}}}}}}$$

For µ_N-N_, we have simply taken the dipole moment of the TS complex in the absence of the alkali. For electric field ɛ_promoter_, we determine from the alkali-induced electrostatic potential plotted along a line perpendicular to the surface through the center of mass of the adsorbate complex.

The promoter-induced electrostatic potential is given by

6 $$\varDelta {\phi }_{{{{{{\rm{promoter}}}}}}}={\phi }_{{{{{{\rm{promoter}}}}}}/{{{{{\rm{M}}}}}}}-{\phi }_{{{{{{\rm{Ru}}}}}}}$$

where *ϕ*_promoter/Ru_ and *ϕ*_Ru_ mean the work function of promoter doped surface and pristine surface. For ɛ_promoter_, we take the slope of ∆*ϕ*_promoter_ at the position of the upper N in the N-N TS.

## Supplementary information

Supplementary Information

## Data Availability

All data needed to evaluate the conclusions are presented in the paper and in Supplementary information file. The datasets generated during and/or analyzed during the current study are available at https://github.com/CatTheoryDTU/spin-effect-data.
