# Supplementary material for: A spin promotion effect in catalytic ammonia synthesis
Source: Nat Commun. 2022 May 2;13:2382. doi: 10.1038/s41467-022-30034-y (PMC9061734; doi:10.1038/s41467-022-30034-y)
Supplement: Supplementary file 1 — Supplementary Information [file 41467_2022_30034_MOESM1_ESM.pdf]

Supplementary Information for

A spin promotion effect in catalytic ammonia synthesis

Ang Cao[1,2], Vanessa J. Bukas[1,2], Vahid Shadravan[1,2], Zhenbin Wang[1,2], Hao Li[1], Jakob Kibsgaard[1], Ib Chorkendorff [1]*, Jens K. Nørskov [1]*

[1] *Department of Physics, Technical University of Denmark, 2800 Kongens Lyngby, Denmark*

[2] These authors contributed equally: Ang Cao, Vanessa J. Bukas, Vahid Shadravan, Zhenbin Wang.

*Corresponding authors, e-mail: ibchork@fysik.dtu.dk, jkno@dtu.dk

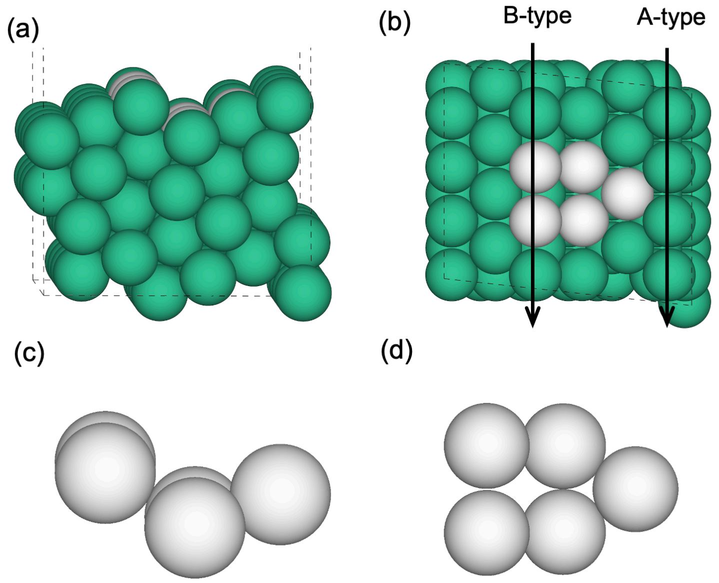

Supplementary Figure 1 The side and top views of the hcp Ru/Co $(10\bar{1}5)$ surface (a-b) and the B5-sites(c-d) in B-type steps. Green spheres represent Ru/Co atoms. An example of the Ru/Co atoms on the B5-sites are labeled in grey. Here two different types of steps are present in the hcp $(10\bar{1}5)$ surface, while we choose the B-type step in our calculations since the B5-site on the B-type step was designated to be the active sites for ammonia synthesis[1], while no B5-site was present on the A-type step.

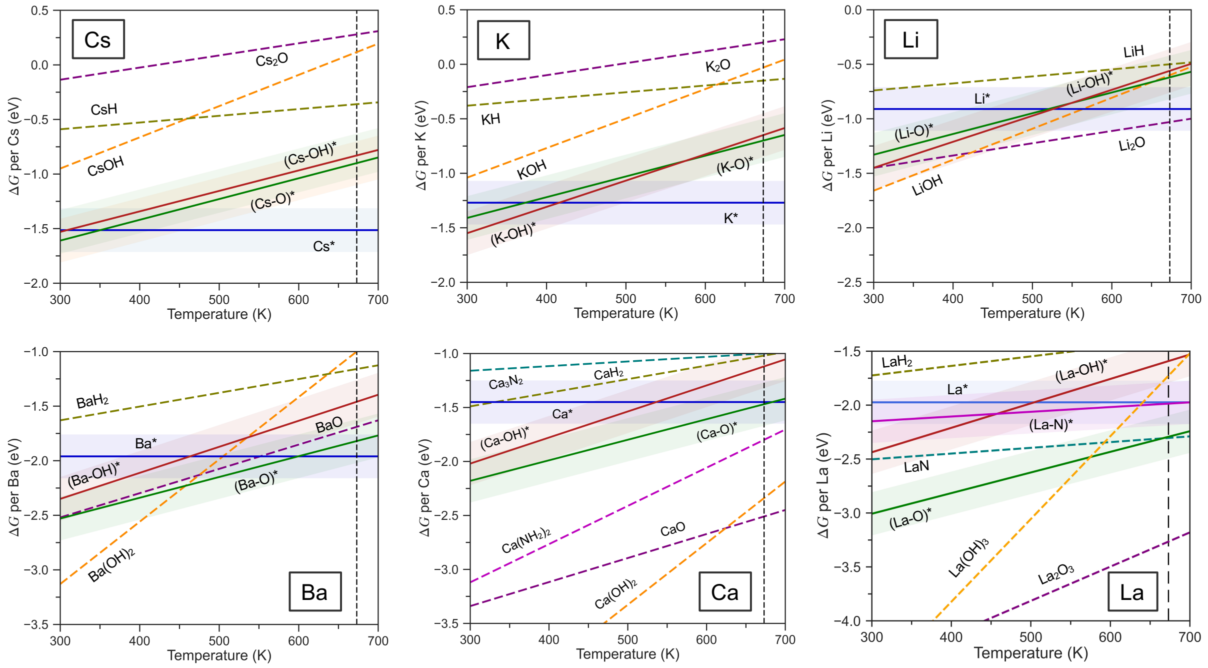

Supplementary Figure 2 The phase diagrams of Cs, K, Li, Ba, Ca and La promoted Co in equilibrium with their oxides, hydroxides hydrides, nitrides, or amides under reaction conditions. The reaction conditions are T=673 K, H_2=7.5 bar, NH_3 = 0.1bar (N_2 conversion of 2%), $P_{H2O}=10^{-7}$ bar.

Compared with the phase diagrams on Ru, it may be somewhat harder to reduce out the precursors in the presence of Co since it binds the precursor atoms a little weaker. But the oxide, hydride, nitride and amide forms of K, Cs, Li, and Ba can still be reduced out of the bulk precursor migrating to the step sites of the Co metal particles under reaction conditions. For Ca and La, it is difficult to reduce the oxide and hydroxides, while in experiments, the used nitride, hydride and amide forms used [2,3] are, however, still reducible at these conditions, if the promoter goes to a step site of the catalyst.

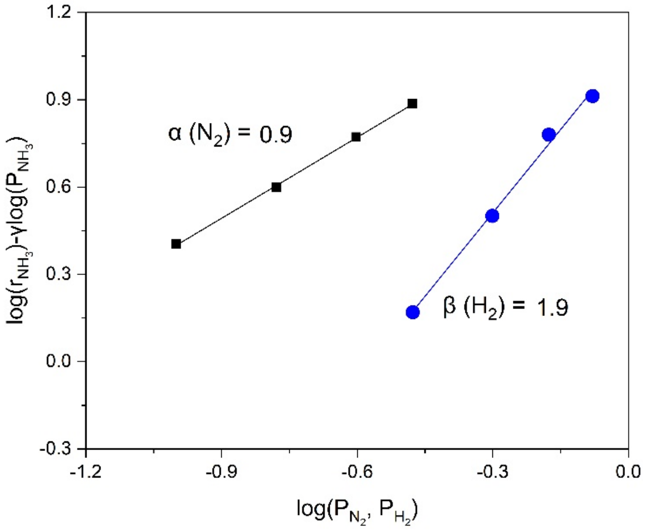

Supplementary Figure 3 The correct calculated reaction orders for Ru/C12A7:e^{-} catalyst reported by M. Kitano et al. [4] based on the data of the original paper for N_2 and H_2.

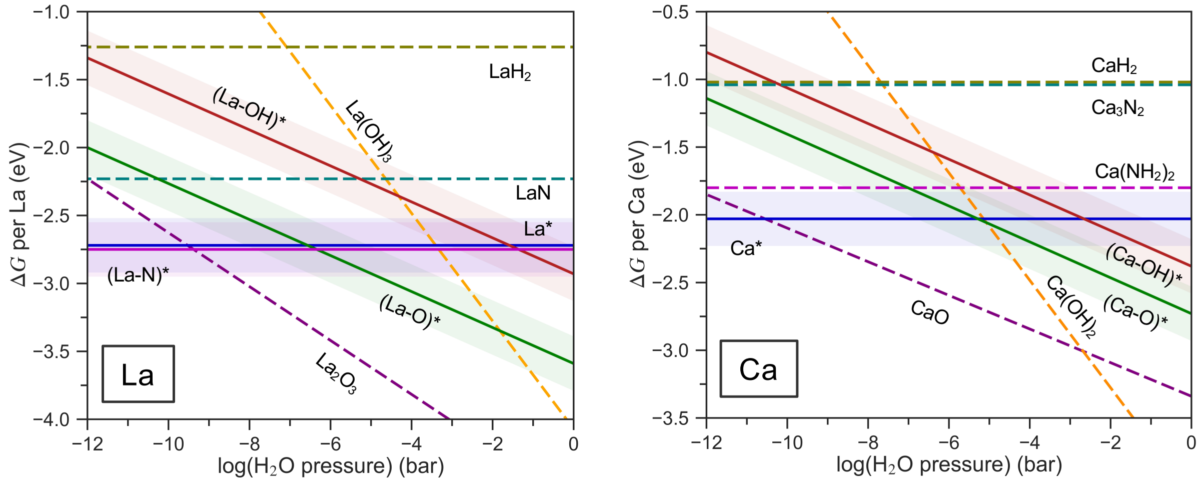

Supplementary Figure 4 The phase diagrams of La and Ca promoted Ru in equilibrium with their oxides, hydroxides hydrides, nitrides as a function of H_2O pressure. Other reaction conditions are T=673 K, H_2=7.5 bar, NH_3 = 0.1bar (N_2 conversion of 2%).

It shows the H_2O pressure has a substantial effect on the reduction of the bulk precursors. If the water content is higher, it becomes more difficult to reduce out the precursor. For La and Ca, it is difficult to reduce the oxide and hydroxides except at the lowest water content considered.

Analysis of electrostatic effect on Ru and Co surfaces

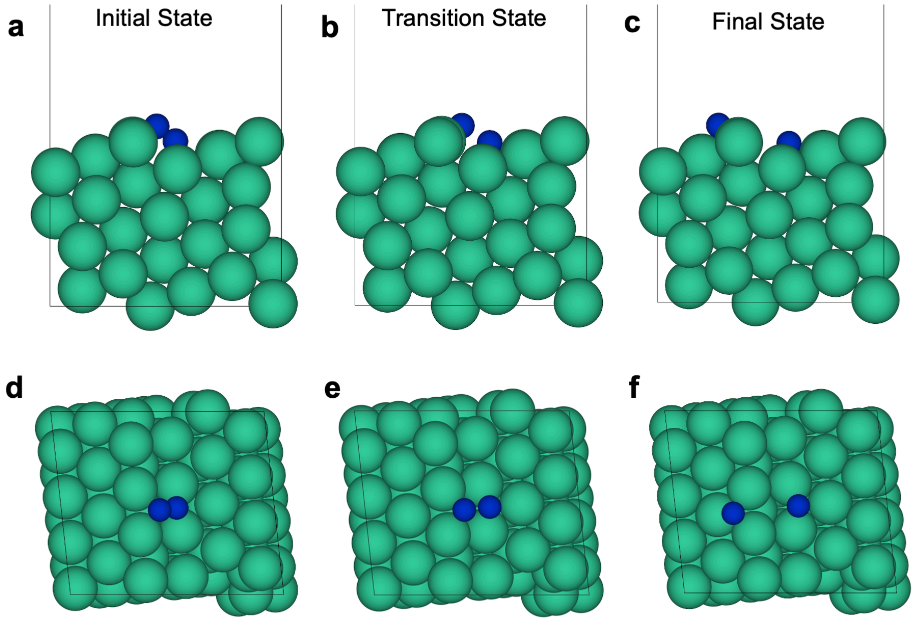

Supplementary Figure 5 The side and top view of the initial state (a, d), transition state (b, e), and final state (d, f) of N_2 dissociation on Ru/Co($10\bar{1}5$) surface. Green and blue spheres represent Ru/Co and N atoms.

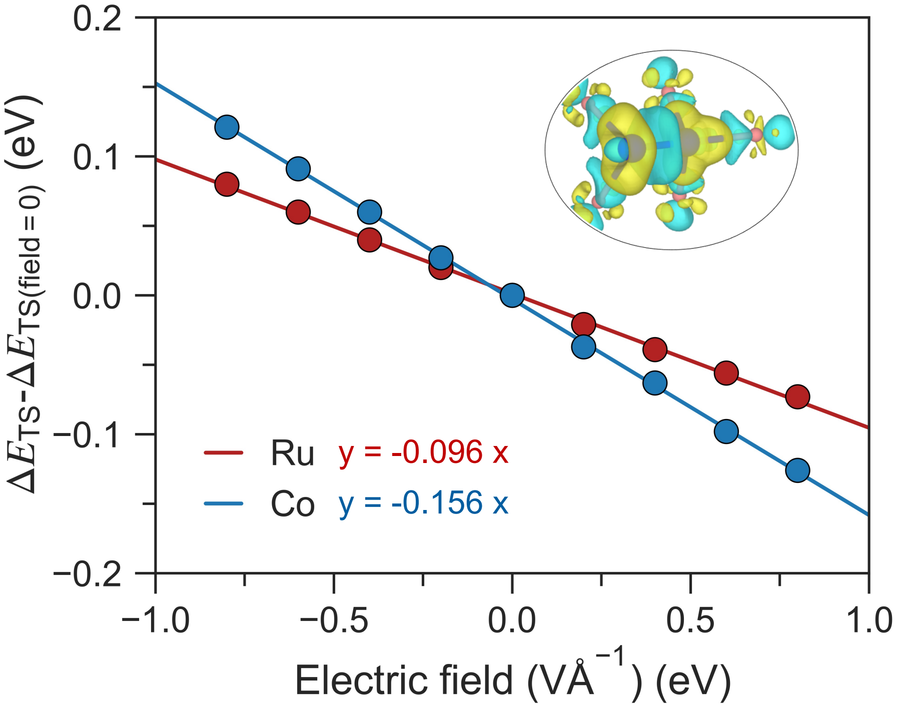

Supplementary Figure 6 Electric field induced variations in stability (relative to ε =0 V/Å) for N-N TS adsorbed at Ru and spin-polarized Co surface. Inset is the charge density difference, where yellow and blue contours represent iso-surfaces of charge accumulation and depletion, respectively.

To measure the N-N transition state (N-N TS) response to the electric field (ε), a second-order polynomial was fitted to calculations for the adsorbate across the range of fields, and values were determined for intrinsic dipole moment (μ) and polarizability (α), using

$\Delta\Delta E_{TS}(\varepsilon) = \Delta E_{TS}(\varepsilon) - \Delta E_{TS}(\varepsilon=0) = \mu\varepsilon - 0.5\ \alpha\varepsilon^2$

where μ_0 is the static surface dipole moment with no applied field, and α is the polarizability of the adsorbate.

As shown in **Supplementary Figure 7**, the second term on the right-hand side of the above equation was negligible, resulting in a ''first-order'' field effect where $\Delta\Delta E_{TS}(\varepsilon)$ varies linearly with ε.

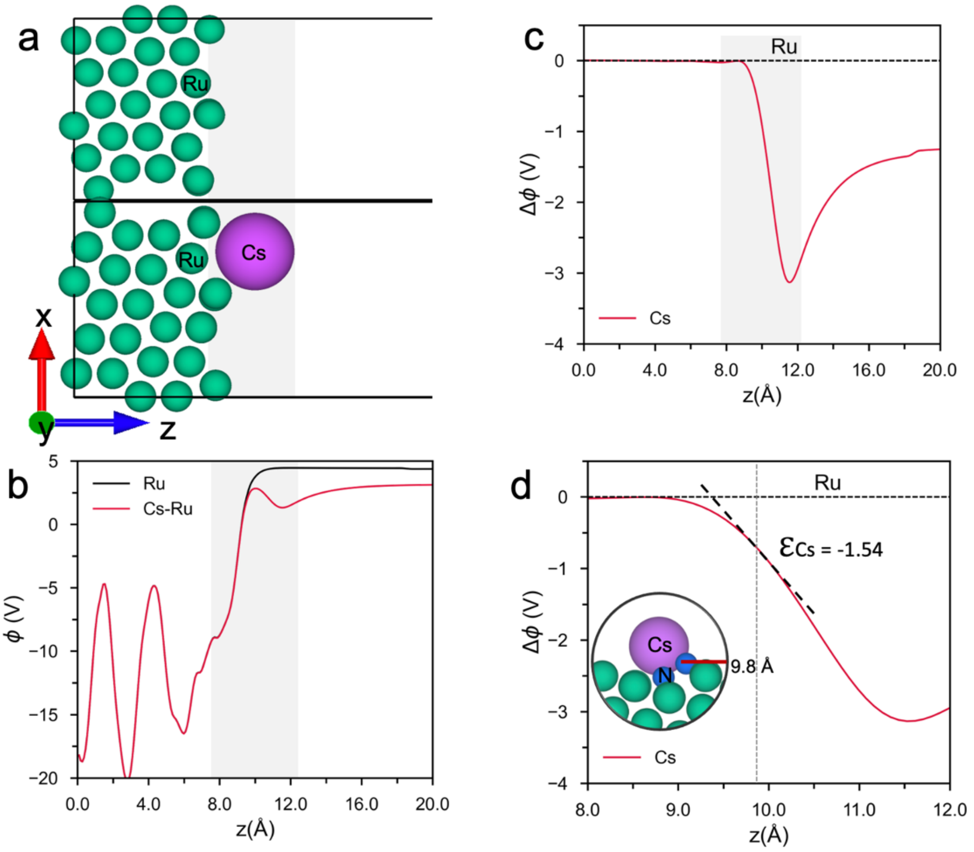

Supplementary Figure 7 Optimized structure of pristine Ru and Cs doped Ru surface (a), the calculated workfunction along the z-axis direction of pristine Ru and Cs doped Ru surface(b), the Cs-induced electrostatic potentials (c-d). Green and purple spheres represent Ru and Cs atoms.

Taking Cs as an example, the calculation procedure of the promoter induced electric field effect is described as follows:

Step 1: Calculate the work function of pristine Ru (ϕ_{Ru}) and Cs doped Ru ($\phi_{promoter/Ru}$) along the z-direction through the region of N-N transition state (Figure b);

Step 2: Calculate the Cs-induced electrostatic potentials based on $\Delta\phi_{Cs} = \phi_{Cs/Ru} - \phi_{Ru}$ (Figure c) and enlarge the region between 8Å and 12 Å where Cs and N-N TS are sitting at (Figure d);

Step 3: Taking the slope at the position of the mass of N-N transition state as the Cs induced electric field.

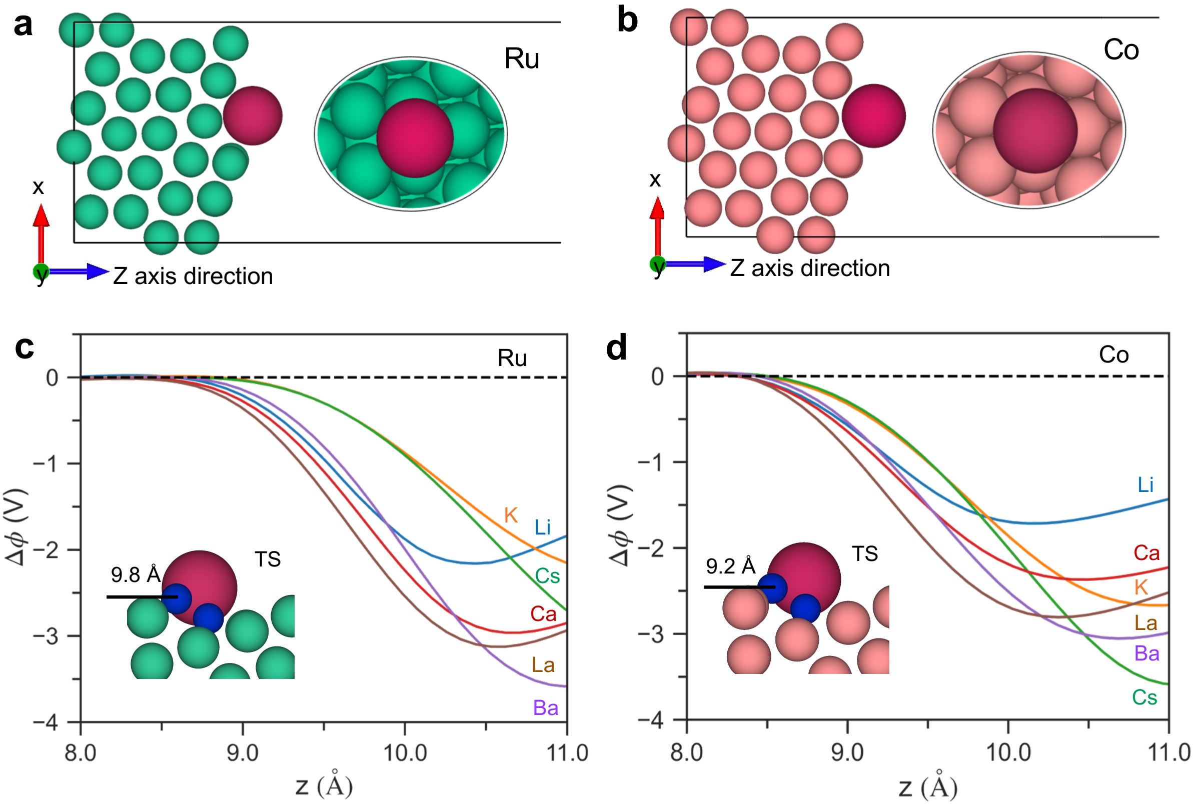

Supplementary Figure 8 Side and top (inset) view of structures of promoter doped Ru($10\bar{1}5$) (**a**) and Co($10\bar{1}5$) surface (**b**), performed on a 4 atom thick layer slab. Green, red and pink spheres represent Ru, promoter and Co atoms. Promoter-induced electrostatic potential plotted along lines perpendicular to the surface (z-axis direction) through the region of the N-N transition state (TS) on Ru ($10\bar{1}5$) (**c**) and Co($10\bar{1}5$) (**d**). The slope at the position of the mass of N-N TS represent promoter induced electric field ($\mathcal{E}_{promoter}$). Insets are the N-N TS on Ru and Co.

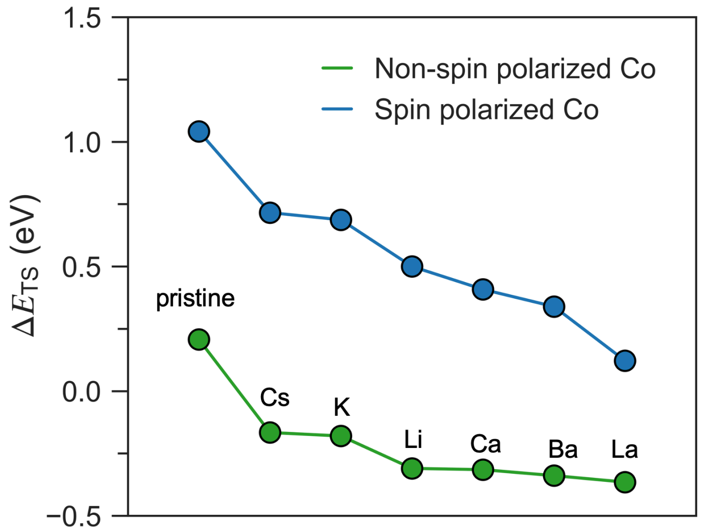

Supplementary Figure 9 N-N TS energy (ΔE_{TS}) on spin polarized and non-spin polarized Co with different promoters. Clearly that the N_2 dissociation barrier is lower on non-spin ploarized surfaces than that on spin polarezed.

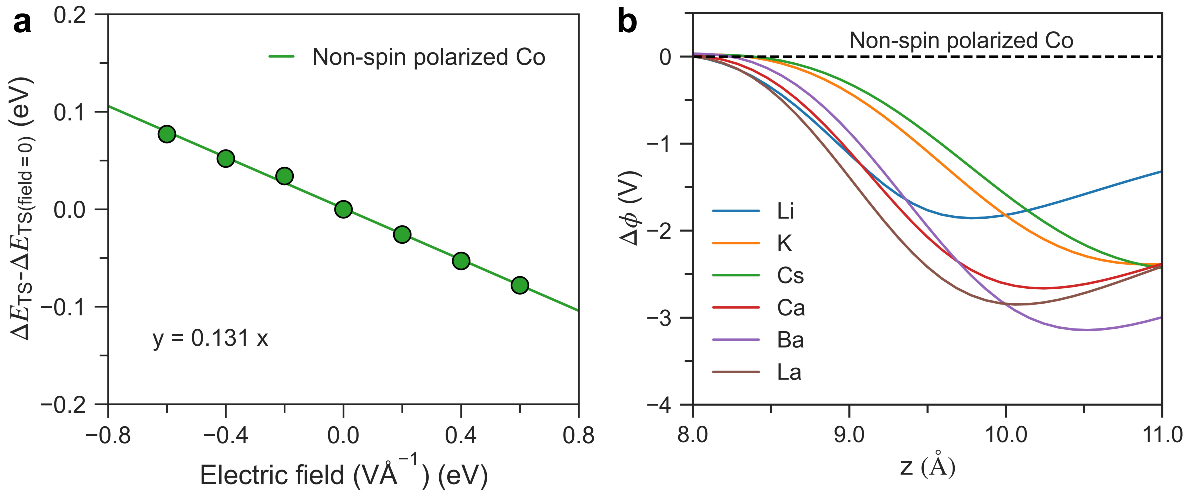

Supplementary Figure 10 a, Electricfield-inducedd variations in stability ($\Delta\Delta G_{TS}$, relative to ε =0 V/Å) for N-N transition state adsorbed at the non-spin polarized Co(1015) surface. **b,** Promoter-induced electrostatic potential plotted along lines perpendicular to the non-spin polarized Co(1015) surface. The slope at the position of upper N in the N-N TS state of 9.2 Å, indicated by the dashed black line representssent the electric field.

To explore the extra effect, the non-spin polarized Co was introduced. As shown in Supplementary Figure 6, the N-N TS energies (ΔE_{TS}) on non-spin polarized Co are much lower than that on spin polarized Co, while the difference in N-N TS energy ($\Delta\Delta E_{TS}$) on spin-polarized Co is more pronounced than on the non-spin polarized Co (**Fig.3b**). Considering that the electrostatic effects ($\Delta E_{promotion}$) are basically the same on spin and non-spin polarized Co (see Supplementary Table 8 and Supplementary Table 9), we identified the extra promotion on spin polarized Co as related to the spin polarization of Co.

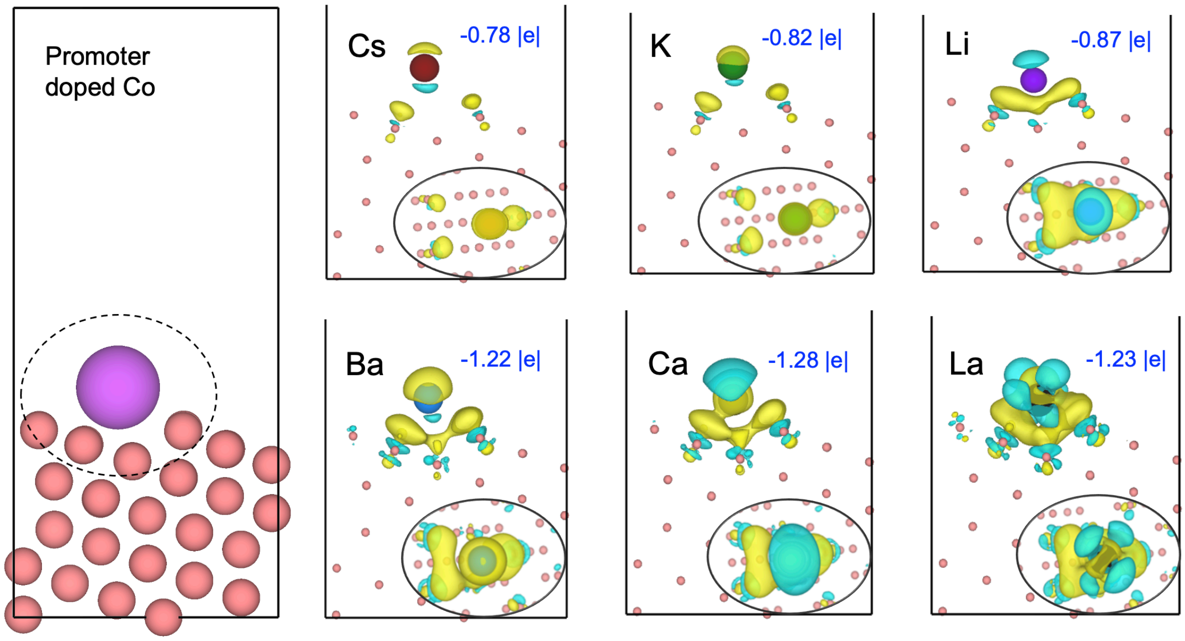

Supplementary Figure 11 Calculated charge density difference of the N-N TS structure over promoter doped Co(1015) surface. Green and purple spheres represent Co, and promoter, respectively. The yellow and blue contours represent isosurfaces of charge accumulation and depletion, respectively. The charge density difference was calculated by $\Delta\rho = \rho_1 - \rho_2 - \rho_3$ (where ρ_1, ρ_2, and ρ_3 represent the charge densities of the whole system, the pristine Co, and the N-N TS adsorbate, respectively). The inset figures are the top view of the promoter doped Co system. The calculated change in electron charge for promoter was labeled in blue, which indicates that the electron will transfer from the promoter to the Co surface.

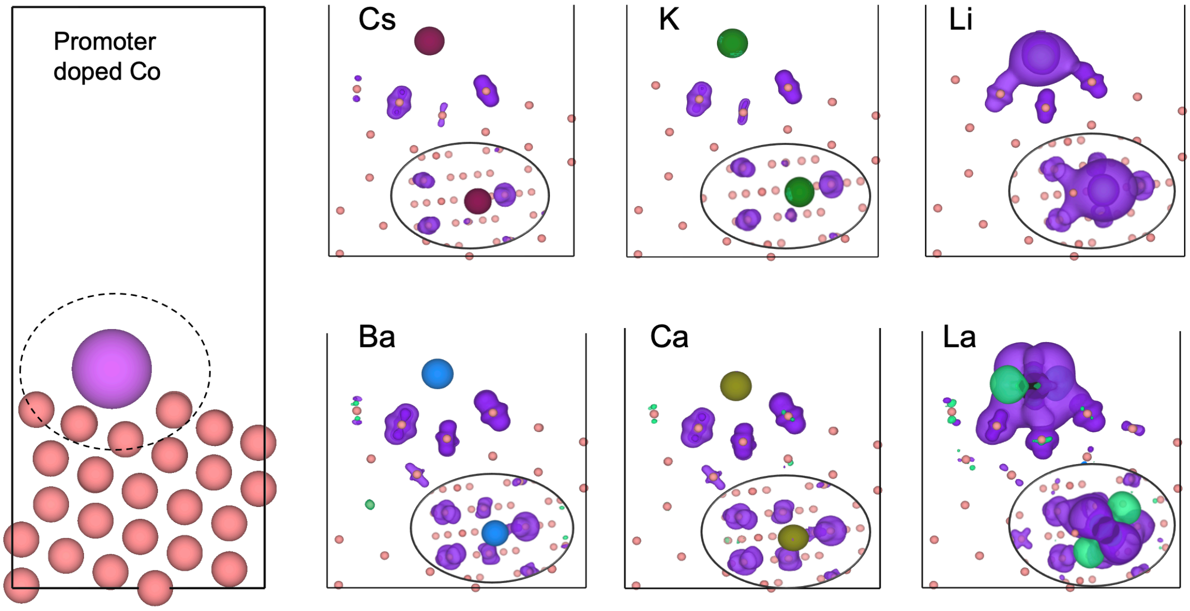

Supplementary Figure 12 Calculated total spin density (including spin up and spin down) difference of the N-N TS structure over promoter doped Co(1015) surface. green, and purple spheres represent Co, and promoter, respectively. The green and purple contours represent isosurfaces of spin density accumulation and depletion, respectively. The spin density difference was calculated by $\Delta\rho = \rho_1 - \rho_2 - \rho_3$ (where ρ_1, ρ_2, and ρ_3 represent the total spin densities of the whole system, the pristine Co, and the N-N TS adsorbate, respectively). The inset figures are the top view of the promoter doped Co system. We can see that the spin density decreases on the atoms around the promoter.

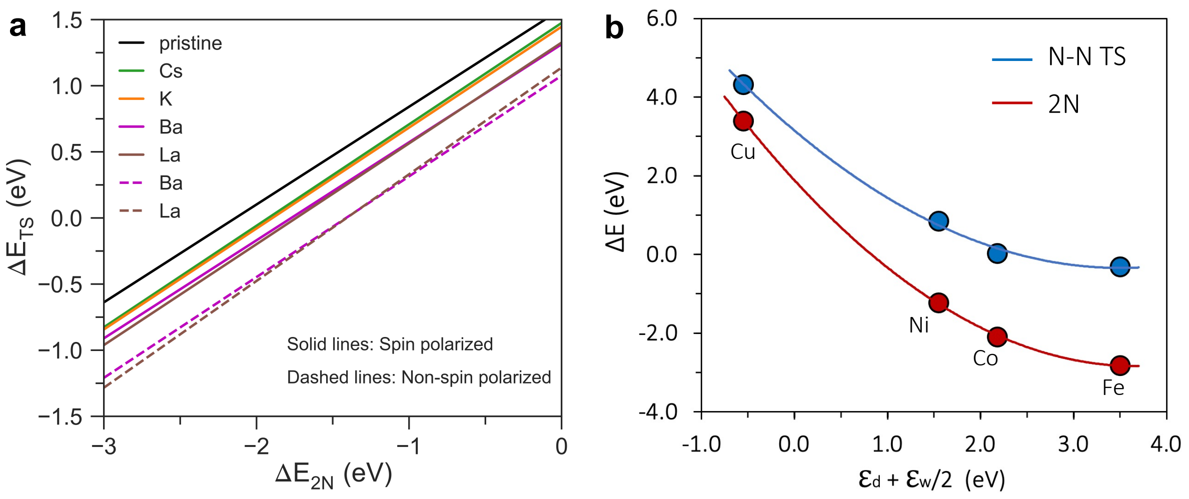

Supplementary Figure 13 a, Scaling relations for ΔE_{TS} as a function of N_2 dissociation energy (ΔE_{2N}) on different promoter doped non-spin polarized (solid lines) and spin polarized (dashed lines) metal surfaces. **b,** Calculated ΔE_{TS} (blue line) and ΔE_{2N} (red line) on different non-spin polarized late 3d metals (Cu, Co, Ni and Fe) as a function of the top of the d-band taken from Ref [5].

We find the spin promotion effect to work for other magnetic metals as well, and the promotional effect of promoters on spin polarized surfaces are larger than the non-spin polarized surfaces, based on the downshift of the scaling lines (dashed line in Figure a). It can be confirmed by Figure b, where the destabilization of ΔE_{2N} is larger than that of ΔE_{TS}, due to a larger curvature for ΔE_{2N} than for ΔE_{TS}.

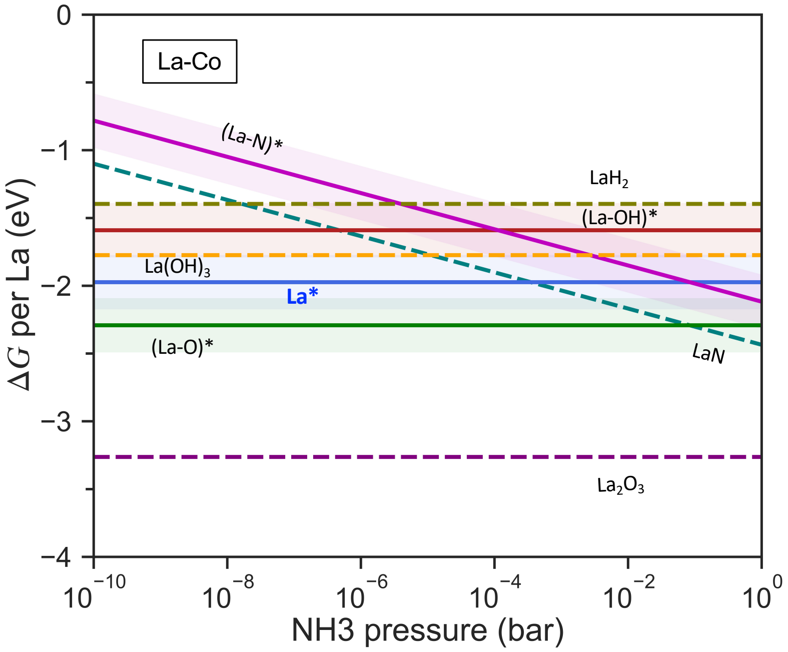

Supplementary Figure 14 Phase diagrams of La promoted Co in equilibrium with their oxides, hydroxides hydrides, nitrides and carbides under reaction conditions as a function of NH_3 pressure. The reaction conditions are T=673 K, H_2=7.5 bar, P_{H2O}=10^{-7} bar, chosen to simulate an extremely dry reactant gas. Bulk species are shown as dashed lines, while adsorbed species are shown as full lines and identified by a*.

Supplementary Table 1. Overview of some of the interesting and recently reported catalysts for thermochemical ammonia synthesis.

Catalyst (as named in the ref.)	Testing conditions		Catalyst Performance	Ref.
	Pressure [bar]	Temperature [˚C]	Activity [$mmol.g^{-1}.h^{-1}$]	
Ba-Ru-Li/AC	10	459	46.3	6
Li-Ru/(111)MgO	10	400	33.04	6
Cs-Ru/(111)MgO	10	400	22	6
Ba-Ru/AC	10	400	8.285	4
Cs-Ru/MgO	10	400	12.117	4
Cs–Ru/r-CeO_2	10	400	14.266	7
Ru/C12A7:e-	10	400	8.245	4
K–Ru/r-CeO_2	10	400	11.227	7
Co/CeO_2-D-500	10	425	19	8
Ru@CeO_2-9	10	425	13.5	8
Cs-Ru/$BaCeO_3$-a (1.25wt%)	10	425	14.57	9
Ru−Ba/Al_2O_3-980	10	400	7.217	10
Ru-Cs/MgO-MIL	10	400	19.2	11
Co–LiH	10	350	11.2	12
Ru/Pr_2O_3	10	400	19.1	13
Ru/$Ce_{0.6}Zr_{0.42}O_2$	10	390	1.7	14
Ru/Ca_2N:e-	10	320	4	15
3BaH_2-10%Co/CNTs	10	400	21	16
Ba-Co/C	10	440	86.4	17
KM1	10	440	46.8	17
LaRuSi after EDTA	10	400	14.3	18
Co/C12A7:e−	10	400	4.2	19
Co-Mo/CeO_2(NaNaph)	10	400	3.15	20
Ru/$Ca(NH_2)_2$	10	320	31.97	21
Ru/Ba-$Ca(NH_2)_2$	10	340	57.05	22
Co/Ba-$Ca(NH_2)_2$	10	380	24.42	22
Ru/BaO−CaH_2	10	320	30.66	22
Ru/Ti-Ce-S	10	400	14.58	23
LaCoSi	10	400	5.5	24
Ru/3LaN/ZrH_2	10	400	12.8	25
Ni/CeN	10	340	9	3
Ni/LaN	10	340	5.3	2
Ru/Ba/$LaCeO_x$	10	400	88.1	26

Supplementary Table 2 Formation energies for per promoter atom in adsorbed species. Experimental formation energy of bulk phase at 300K from ref [27]

	Adsorbates	Li*	(LiO)*	(LiOH)*	Cs*	(CsO)*	(CsOH)*
Surfaces	Ru	-1.20	-1.96	-2.24	-1.74	-2.27	-2.51
	Co	-0.91	-1.73	-2.10	-1.51	-2.01	-2.37
	Adsorbates	Ba*	(BaO)*	(BaOH)*	Ca*	(CaO)*	(CaOH)*
Surfaces	Ru	-2.45	-3.27	-3.33	-2.02	-2.91	-2.99
	Co	-1.96	-2.93	-3.00	-1.45	-2.67	-2.73
	Adsorbates	La*	(LaO)*	(LaN)*	K*	(KO)*	(KOH)*
Surfaces	Ru	-2.72	-3.77	-2.65	-1.51	-2.10	-2.24
	Co	-1.97	-3.32	-1.58	-1.27	-1.81	-2.19

Supplementary Table 3 ZPE, enthalpic temperature correction, and *S* of gas-phase.

Species	ZPE (eV)	C_p (eV/K)	S (eV/K)
H_2	0.273	0.000305215	0.00136125
H_2O	0.584	0.000355126	0.001967031
N_2	0.146	0.000305215	0.001995927
NH3	0.923	0.000349105	0.002008021

Supplementary Table 4 ZPE, enthalpic temperature correction, and *S* of adsorbates calculated using the RPBE functional.

Temperature (K)	adsorbates	ZPE (eV)	$\int C_P \mathrm{d}T$ (eV)	S (eV/K)
298	H*	0.16	0.01	0.0000645
	O*	0.06	0.03	0.0002037
	OH*	0.35	0.05	0.0002921
	N*	0.09	0.02	0.0000932
673	H*	0.160	0.069	0.0001809
	O*	0.063	0.120	0.0003892
	OH*	0.352	0.188	0.0005877
	N*	0.089	0.100	0.0002639

Supplementary Table 5 N adsorption energy (ΔE_N) and N-N transition state energies (ΔE_{TS}) on pristine and promoter doped metal surfaces.

surfaces		Pristine (eV)	Cs (eV)	K (eV)	Li (eV)	Ba (eV)	Ca (eV)	La (eV)
Ru	ΔE_N	-0.85	-0.88	-0.88	-	-0.91	-0.91	-0.92
	ΔE_{TS}	0.169	-0.007	-0.006	-0.004	-0.122	-0.101	-0.119
Rh	ΔE_N	-0.398	-0.443	-0.443	-	-0.459	-	-0.445
	ΔE_{TS}	0.998	0.870	0.842	-	0.793	-	0.829
Pd	ΔE_N	0.541	0.534	0.537	-	0.571	-	0.655
	ΔE_{TS}	2.343	2.247	2.225	-	2.231	-	2.273
Spin polarized Co	ΔE_N	-0.282	-0.300	-0.326	-	-0.437	-0.459	-0.633
	ΔE_{TS}	1.042	0.716	0.687	0.524	0.408	0.339	0.122
Non-spin polarized Co	ΔE_N (eV)	-1.045	-1.082	-1.075	-	-1.080	-1.068	-1.068
	ΔE_{TS}	0.027	-0.166	-0.180	-0.309	-0.315	-0.339	-0.365
Spin polarized Ni	ΔE_N (eV)	-0.15	-0.10	-	-	-0.03	-	-0.19
	ΔE_{TS}	1.677	1.244	1.218	-	1.033	-	0.826
Non-spin polarized Ni	ΔE_N (eV)	-0.61	-0.60	-0.58	-	-0.57	-	-0.57
	ΔE_{TS}	0.856	0.638	0.618	-	0.496	-	0.427

Supplementary Table 6 The calculated electric field (ε), $\Delta E_{promotion}$, and DFT calculated $\Delta\Delta E_{TS}$ (eV) by NEB method on promoter doped Ru surface.

promoter	Li	K	Cs	Ca	Ba	La
electric field ε (V/Å)	-1.67	-1.54	-1.54	-2.5	-2.67	-2.58
$\Delta E_{promotion}$ (eV)	-0.16	-0.15	-0.15	-0.24	-0.26	-0.25
calculated $\Delta\Delta E_{TS}$ (eV)	-0.17	-0.18	-0.17	-0.27	-0.29	-0.29

Supplementary Table 7 The calculated electric field (ε), $\Delta E_{\mathrm{promotion}}$, and DFT calculated $\Delta\Delta E_{TS}$ (eV) by NEB method on promoter doped spin polarized Co surface.

promoter	K	Cs	Li	Ba	Ca	La
electric field ε (V/Å)	-1.67	-1.53	-1.88	-2.05	-2.0	-2.25
$\Delta E_{\mathrm{promotion}}$ (eV)	-0.26	-0.24	-0.29	-0.32	-0.31	-0.35
Calculated $\Delta\Delta E_{TS}$ (eV)	-0.35	-0.33	-0.52	-0.63	-0.70	-0.92

Supplementary Table 8 The calculated electric field (ε), $\Delta E_{\mathrm{promotion}}$, and DFT calculated $\Delta\Delta E_{TS}$ (eV) by NEB method on promoter doped non-spin polarized Co surface.

promoter	K	Cs	Li	Ba	Ca	La
electric field ε (V/Å)	-1.43	-1.25	-2.0	-2.38	-2.43	-2.5
$\Delta E_{\mathrm{promotion}}$ (eV)	-0.19	-0.16	-0.26	-0.32	-0.32	-0.33
Calculated $\Delta\Delta E_{TS}$ (eV)	-0.21	-0.19	-0.33	-0.34	-0.37	-0.40

Supplementary Table 9 The difference in the magnetic moment (Δ(mag)) of Co atoms at the B5-sites between the pristine and promoter doped Co.

Magnetic Moment difference	Atom index	Cs	K	Li	Ba	Ca	La
B5-sites	1	-0.017	-0.023	0.006	-0.066	-0.103	-0.168
	2	-0.052	-0.054	0.008	-0.125	-0.146	-0.210
	3	0.001	0.001	0.000	0.001	0.001	0.003
	4	-0.016	-0.014	0.001	-0.016	-0.010	-0.010
	5	-0.030	-0.027	0.004	-0.063	-0.068	-0.097
Δ(mag)		-0.114	-0.117	-0.188	-0.269	-0.326	-0.482

Supplementary Table 10 The activity of ammonia synthesis (r_{NH3}) and free energy barriers (ΔG_{TS}) for different promoters with various metal (Ru, Ni and Co) catalysts at the temperature of 623K and the total pressure of 10bar.

Promoter-TM	Catalyst	r_{NH3} ($mmol.g^{-1}.h^{-1}$)	Ln(r_{NH3})	Refs	ΔG_{TS} (eV)
Cs-Ru	RuCs/C	11.45	2.44	This work	0.94
Ca-Ru	Ru/$Ca(NH_2)_2$	34	3.53	Ref[21]	0.85
La-Ru	Ru/LaN	11.6	2.45	Ref[2]	0.83
Ba-Co	Co-Ba/C	25.2	3.23	Ref[17]	1.36
Cs-Co	Co-Cs/MgO	0.05	-3	Ref[28]	1.67
Ca-Co	Co/C12A7:e− ($12CaO\cdot 7Al_2O_3$)	1.05	0.05	Ref[28]	1.29
Li-Co	Co-LiH	11.5	2.44	Ref[12]	1.49
La-Co	Co/LaN	9.7	2.27	Ref[2]	1.07
Ba-Ni	NiBa/C	0.13	-2.04	Ref[17]	1.98
Li-Ni	Ni-LiH	0.4	-0.92	Ref[12]	2.10
La-Ni	Ni/LaN	7.3	1.99	Ref[2]	1.78

Supplementary References

1. Dahl, S., Taylor, P. A., Törnqvist, E. & Chorkendorff, I. The synthesis of ammonia over a ruthenium single crystal. *J. Catal.* **178**, 679–686 (1998).
2. Ye, T. N. *et al.* Vacancy-enabled N2 activation for ammonia synthesis on an Ni-loaded catalyst. *Nature* **583**, 391–395 (2020).
3. Ye, T. N. *et al.* Contribution of Nitrogen Vacancies to Ammonia Synthesis over Metal Nitride Catalysts. *J. Am. Chem. Soc.* **142**, 14374–14383 (2020).
4. Kitano, M. *et al.* Ammonia synthesis using a stable electride as an electron donor and reversible hydrogen store. *Nat. Chem.* **4**, 934–940 (2012).
5. Medford, A. J. *et al.* From the Sabatier principle to a predictive theory of transition-metal heterogeneous catalysis. *J. Catal.* **328**, 36–42 (2015).
6. Zheng, J. *et al.* Efficient Non-dissociative Activation of Dinitrogen to Ammonia over Lithium-Promoted Ruthenium Nanoparticles at Low Pressure. *Angew. Chemie Int. Ed.* **58**, 17335–17341 (2019).
7. Ma, Z., Zhao, S., Pei, X., Xiong, X. & Hu, B. New insights into the support morphology-dependent ammonia synthesis activity of Ru/CeO2 catalysts. *Catal. Sci. Technol.* **7**, 191–199 (2017).
8. Wang, X. *et al.* Efficient ammonia synthesis over a core-shell Ru/CeO 2 catalyst with a tunable CeO 2 size: DFT calculations and XAS spectroscopy studies. *Inorg. Chem. Front.* **6**, 396–406 (2019).
9. Li, W., Wang, S. & Li, J. Highly Effective Ru/BaCeO $_3$ Catalysts on Supports with Strong Basic Sites for Ammonia Synthesis. *Chem. – An Asian J.* **14**, asia.201900618 (2019).
10. Lin, B. *et al.* Ammonia Synthesis Activity of Alumina-Supported Ruthenium Catalyst Enhanced by Alumina Phase Transformation. *ACS Catal.* **9**, 1635–1644 (2019).
11. Li, J. *et al.* Sub-nm ruthenium cluster as an efficient and robust catalyst for decomposition and synthesis of ammonia: Break the "size shackles".

Nano Res. **11**, 4774–4785 (2018).

12. Wang, P. *et al.* Breaking scaling relations to achieve low-temperature ammonia synthesis through LiH-mediated nitrogen transfer and hydrogenation. *Nat. Chem.* **9**, 64–70 (2017).
13. Sato, K. *et al.* A low-crystalline ruthenium nano-layer supported on praseodymium oxide as an active catalyst for ammonia synthesis. *Chem. Sci.* **8**, 674–679 (2017).
14. Ma, Z., Xiong, X., Song, C., Hu, B. & Zhang, W. Electronic metal-support interactions enhance the ammonia synthesis activity over ruthenium supported on Zr-modified CeO2 catalysts. *RSC Adv.* **6**, 51106–51110 (2016).
15. Kitano, M. *et al.* Essential role of hydride ion in ruthenium-based ammonia synthesis catalysts. *Chem. Sci.* **7**, 4036–4043 (2016).
16. Gao, W. *et al.* Barium Hydride-Mediated Nitrogen Transfer and Hydrogenation for Ammonia Synthesis: A Case Study of Cobalt. *ACS Catal.* **7**, 3654–3661 (2017).
17. Hagen, S. *et al.* New efficient catalyst for ammonia synthesis: Barium-promoted cobalt on carbon. *Chem. Commun.* **11**, 1206–1207 (2002).
18. Li, J. *et al.* Acid-durable electride with layered ruthenium for ammonia synthesis: Boosting the activity via selective etching. *Chem. Sci.* **10**, 5712–5718 (2019).
19. Inoue, Y. *et al.* Direct Activation of Cobalt Catalyst by 12CaO·7Al2O3 Electride for Ammonia Synthesis. *ACS Catal.* **9**, 1670–1679 (2019).
20. Tsuji, Y. *et al.* Ammonia synthesis over Co-Mo alloy nanoparticle catalyst prepared via sodium naphthalenide-driven reduction. *Chem. Commun.* **52**, 14369–14372 (2016).
21. Inoue, Y. *et al.* Efficient and Stable Ammonia Synthesis by Self-Organized Flat Ru Nanoparticles on Calcium Amide. *ACS Catal.* **6**, 7577–7584 (2016).

22. Kitano, M. *et al.* Self-organized Ruthenium–Barium Core–Shell Nanoparticles on a Mesoporous Calcium Amide Matrix for Efficient Low-Temperature Ammonia Synthesis. *Angew. Chemie - Int. Ed.* **57**, 2648–2652 (2018).
23. Wu, Y. *et al.* Enhanced ammonia synthesis performance of ceria-supported Ru catalysts: Via introduction of titanium. *Chem. Commun.* **56**, 1141–1144 (2020).
24. Gong, Y. *et al.* Ternary intermetallic LaCoSi as a catalyst for N2 activation. *Nat. Catal.* **1**, 178–185 (2018).
25. Li, L. *et al.* Operando spectroscopic and isotopic-label-directed observation of LaN-promoted Ru/ZrH2 catalyst for ammonia synthesis via associative and chemical looping route. *J. Catal.* **389**, 218–228 (2020).
26. Sato, K. *et al.* Surface dynamics for creating highly active ru sites for ammonia synthesis: Accumulation of a low-crystalline, oxygen-deficient nanofraction. *ACS Sustain. Chem. Eng.* **8**, 2726–2734 (2020).
27. Rumpf, D.-I. B. *Thermochemical data of pure substances. Veterinary Immunology and Immunopathology* vol. 55 (1997).
28. Inoue, Y. *et al.* Direct Activation of Cobalt Catalyst by 12CaO·7Al 2 O 3 Electride for Ammonia Synthesis. *ACS Catal.* **9**, 1670–1679 (2019).
